# Synthesis, Crystal Structure, and Thermal Decomposition of the Cobalt(II) Complex with 2-Picolinic Acid

**DOI:** 10.1155/2014/641608

**Published:** 2014-01-20

**Authors:** Di Li, Guo-Qing Zhong

**Affiliations:** State Key Laboratory Cultivation Base for Nonmetal Composite and Functional Materials, School of Material Science and Engineering, Southwest University of Science and Technology, Mianyang 621010, China

## Abstract

The cobalt(II) complex of 2-picolinic acid (Hpic), namely, [Co(pic)_2_(H_2_O)_2_] · 2H_2_O, was synthesized with the reaction of cobalt acetate and 2-picolinic acid as the reactants by solid-solid reaction at room temperature. The composition and structure of the complex were characterized by elemental analysis, infrared spectroscopy, single crystal X-ray diffraction, and thermogravimetry-differential scanning calorimetry (TG-DSC). The crystal structure of the complex belongs to monoclinic system and space group *P*2(1)/*n*, with cell parameters of *a* = 9.8468(7) Å, *b* = 5.2013(4) Å, *c* = 14.6041(15) Å, *β* = 111.745(6)°, *V* = 747.96(11) Å^3^, *Z* = 2, *D*_*c*_ = 1.666 g cm^−3^, *R*_1_ = 0.0297, and *wR*_2_ = 0.0831. In the title complex, the Co(II) ion is six-coordinated by two pyridine N atoms and two carboxyl O atoms from two 2-picolinic acid anions, and two O atoms from two H_2_O molecules, and forming a slightly distorted octahedral geometry. The thermal decomposition processes of the complex under nitrogen include dehydration and pyrolysis of the ligand, and the final residue is cobalt oxalate at about 450°C.

## 1. Introduction

In recent years, chemists have tended to design and synthesise metal-organic frameworks, which are undergoing accelerated and sustained growth because of their fascinating structures and potential applications, such as molecular adsorption, catalysis, gas storage, multifunctional materials, and chemical separation [1–10]. The transition metal carboxylates have the diversity of coordination modes for carboxyl group, which tend to form cluster or polymer structures [11], and it is significance to synthesis of some specific structure and function complexes in future. It is known that 2-picolinic acid is terminal tryptophan metabolite, and its anion has been used as a valuable chelating ligand, and the pyridine N atom and the carboxylate oxygen atoms are capable of coordinating various metal ions. Besides, 2-picolinic acid is not only a potential proton acceptor but also proton donor depending on deprotonated groups [12]. To the best of our knowledge, the crystal structures of 2-picolinic acid with Ni(II), Zn(II), Co(III), and Cu(II) derivations have been reported [11–14].

The method of room temperature solid-solid synthesis is a simple and convenient method for the preparation of metal complexes, and many complexes have been synthesized by this method [15–17]. Advantages of the solid-solid synthesis method are much higher yield, being inexpensive, faster reaction rate, easier operating, energy saving, and environmental friendly [18], and it is in accordance with the requirements of green chemistry. Cobalt is an essential trace element for human and all animals, and its complexes have been used in the fields of medicine, bioinorganic chemistry, functional materials, and so forth [19–24]. At the same time, the cobalt compounds are often used in chemical reactions as oxidation catalysts, such as typical catalysts that are the cobalt carboxylates, which are also used in paints, varnishes, and pigments industry [25]. We report herein the X-ray single crystal structure and thermal property of the cobalt(II) complex of 2-picolinic acid which is synthesized by solid-solid reaction at room temperature.

## 2. Experimental

### 2.1. Materials and Physical Measurements

All the chemicals used in the experiments were analytical reagents as received from commercial sources and without further purification. 2-Picolinic acid was purchased from Alfa Aesar, while cobalt(II) acetate tetrahydrate was purchased from Merck.

The contents of carbon, hydrogen, and nitrogen in the complex were measured by a Vario EL CUBE elemental analyzer, and the cobalt content was determined by EDTA complexometric titration. The FTIR spectra were obtained with a Perkin-Elmer Spectrum One-Spectrometer in the ranges of 400–4000 cm^−1^ using KBr pellets. The thermogravimetric analysis of the metal complex was performed by a SDT Q600 thermogravimetric analyzer, and the measurement was recorded from 30 to 600°C at the heating rate of 10°C min^−1^ under nitrogen flow of 50 mL min^−1^. X-ray powder diffraction was performed using a D/max-II X-ray diffractometer, Cu *K*_*α*_ radiation (*λ* = 0.154056 nm, step width: 2*θ* = 0.2°, and scan speed: 8°/min).

### 2.2. Synthesis of the Complex [Co(pic)_2_(H_2_O)_2_]·2H_2_O

The synthesis reaction of the title complex is as follows:

(1) 2C6H5O2N+Co(CH3COO)2·4H2O  ⟶  [Co(C6H4O2N)2(H2O)2]·2H2O    +2CH3COOH↑

2-Picolinic acid and cobalt acetate of the two reactants were weighed and placed in an agate mortar, and the molar ratio of 2-picolinic acid to cobalt acetate was 2 : 1. Then, the mixture was grinded carefully at room temperature and released a strong irritant gas in the grinding process. The released gas was tested with moist pH paper, and the result indicated that the gas was faintly acid gas. The reason was that the acetic acid was released in the reaction process. When there was no irritant gas that escaped, the reaction was completed. The reaction was conducted in grinding at room temperature for 6 h, and the mixture became into loose pink powder. Afterwards the resultant was transferred to the beaker and stirred to dissolve with a little distilled water, and the solution was filtered and concentrated. The concentrated solution was placed at room temperature about 10 days, and the red-orange crystals of the cobalt(II) complex were obtained. The yield of the complex was about 82%. Anal. Calcd. for CoC_12_H_16_O_8_N_2_ (%): C, 38.40; H, 4.27; N, 7.47; Co, 15.71. Found (%): C, 38.26; H, 4.23; N, 7.41; Co, 15.53.

### 2.3. X-Ray Diffraction Crystallography

The appropriate crystal was cut from larger crystals and mounted on a Bruker Smart Apex II CCD diffractometer with graphite monochromated Mo *K*_*α*_ radiation (*λ* = 0.71073 Å). The data were collected at 298(2) *K* using multiscan modes. A red-orange crystal with dimensions 0.42 mm × 0.35 mm × 0.25 mm was mounted on a glass fiber. Diffraction data were collected in *ω* mode in the ranges of 2.50°–25.02°. The programs SHELXS-97 and SHELXL-97 were used for the structure determination and refinement [26, 27]. The structure was solved by direct methods, and all nonhydrogen atoms were obtained from the difference Fourier map and subjected to anisotropic refinement by full-matrix least squares on *F* ^2^. All nonhydrogen atoms were refined anisotropically. The structure refinement parameters for the title complex are given in Table 1, and the crystallographic data are deposited with the Cambridge Crystallographic Data Centre under deposition number CCDC 913361.

## 3. Results and Discussion

### 3.1. X-Ray Crystal Structure Analysis

**Figure 1:**
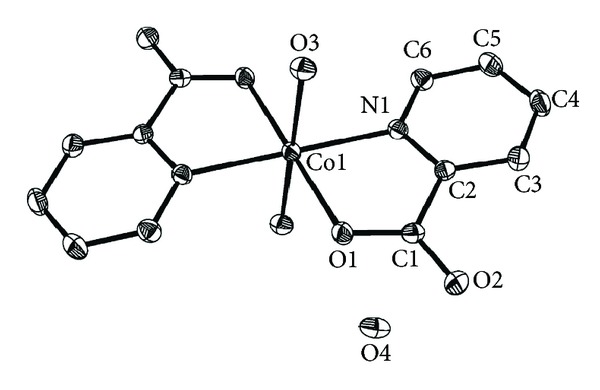
Molecular structure of the title complex.

**Figure 2:**
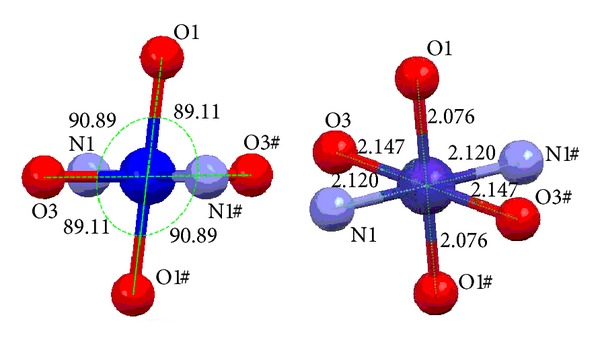
Coordination environment of the Co(II) ion.

Figure 1 shows the key fragments of the structures and the atom numbering in the title complex. Crystallographic data and structure refinement parameters for the title complex are given in Table 1, and the selected bond distances and angles are shown in Table 2. The unit of the complex is composed of one Co(II) ion, two 2-picolinic acid anions, and four water molecules. The Co(II) ion occupies the center of symmetry, which is six-coordinated through two nitrogen atoms and two hydroxy oxygen atoms of the carboxyl group [Co(1)–N(1), 2.1196(18) Å; Co(1)–N(1)#1, 2.1196(18) Å; and Co(1)–O(1), 2.0765(15) Å; Co(1)–O(1)#1, 2.0765(15) Å] from two 2-picolinic acid anions and two oxygen atoms from two H_2_O molecules [Co(1)–O(3), 2.1477(16) Å; and Co(1)–O(3)#1, 2.1477(16) Å]. The complex is formed by 2-picolinic acid anion as bidentate ligand and the space group is *P*2(1)/*n*. The O or N atoms of coordination from the two 2-picolinic acid molecules are equivalent.  Figure 2 shows that the four atoms of O(1), N(1), O(1)#1, and N(1)#1 are in the equatorial plane and form a parallelogram array, and the bond angle of O(3)#1–Co(1)–O(3) is 180° and the atoms of O(3)#1, Co(1), and O(3) are in a straight line; therefore the two atoms of O(3) and O(3)#1 from the coordinated water molecules are in the axial symmetry position, forming a slightly distorted octahedral geometry. In the complex, the bond length of Co(1)–O(1) is slightly shorter than the bond lengths of Co(1)–N(1) and Co(1)–O(3); this indicates that the coordination ability of the carboxyl O atom is stronger than that of the N atom from pyridine ring and the O atom from water molecule. The bond angles (O(1)–Co(1)–O(3) and O(1)#1–Co(1)–O(3)#1, 90.89(6)°; O(1)#1–Co(1)–O(3) and O(1)–Co(1)–O(3)#1, 89.11(6)°) in the complex are near to ideal 90° values. Because of the Jahn-Teller effect of the Co(II) ion with d^7^ electron configuration, the axial bond lengths of Co(1)–O(3) and Co(1)–O(3)#1 are stretched.

**Figure 3:**
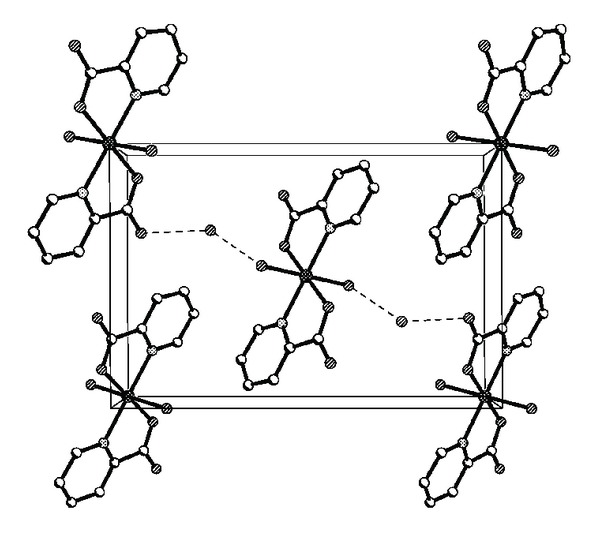
Crystal packing diagram of the title complex.

**Figure 4:**
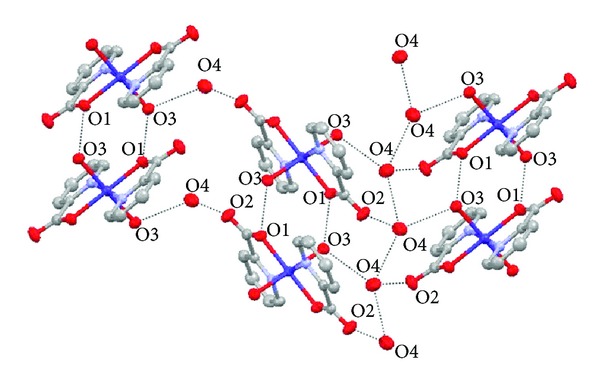
Packing diagram of the title complex showing H bonding.

**Figure 5:**
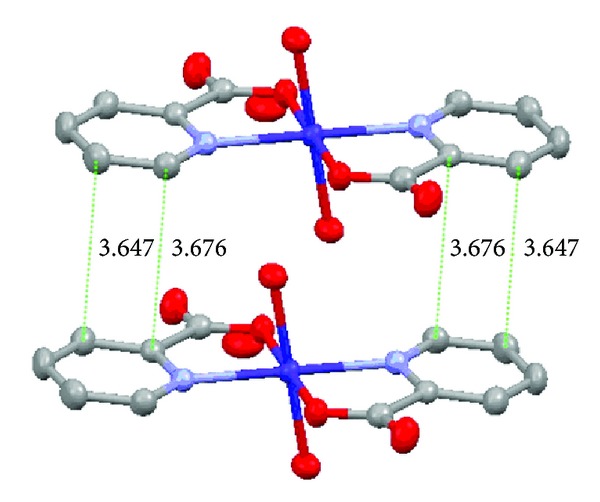
Weak spatial *π*-*π* stacking interactions of the title complex.

In Figures 3 and 4, the molecules of the complex [Co(pic)_2_(H_2_O)_2_] · 2H_2_O are held together by intermolecular hydrogen bonds. Hydrogen bond length and bond angle for the title complex are given in Table 3. There are three types of hydrogen bonds in the crystal of the Co(II) complex, and they are the weak hydrogen bonds between the crystalline water molecules (O4–H4D*⋯*O4 and O4–H4B*⋯*O4, 2.943 Å), the hydrogen bonds between the crystalline water and the coordinated water molecules (O3–H3D*⋯*O4, 2.808 Å), and the strong hydrogen bonds between the oxygen atoms of the carboxylate groups with coordinated and crystallization water molecules (O3–H3C*⋯*O1, 2.739 Å; and O4–H4C*⋯*O1, 2.700 Å), respectively. Hydrogen bonds exist between the crystalline water and the coordinated water molecules, which form a 1D zigzag polymeric chain. At the same time the hydrogen bonds between the carboxyl oxygen atoms and the coordinated water molecules from the different complex molecules form 2D layers; as a result, these 2D layers are extended through extensive hydrogen bonding interactions to form an infinite 3D supramolecular network, and make the molecular structure more stable. Besides, the distances between pyridine rings situating in neighbouring mirror planes are 3.647 Å and 3.676 Å in Figure 5, which may indicate the existence of face-to-face *π*-*π* stacking weak interactions in the complex [28].

### 3.2. FT-IR Spectra

**Figure 6:**
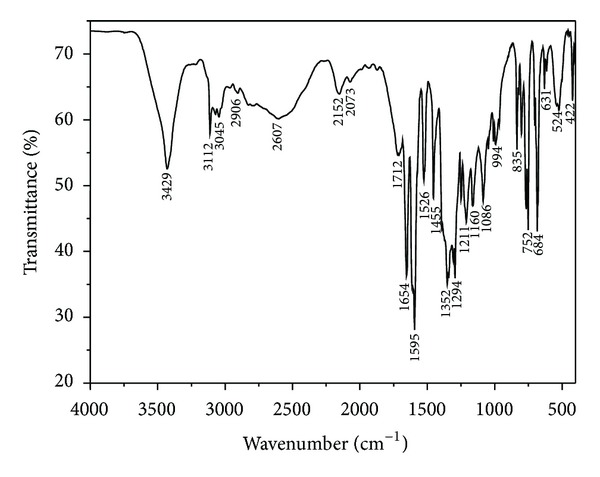
FT-IR spectra of the ligand 2-picolinic acid.

**Figure 7:**
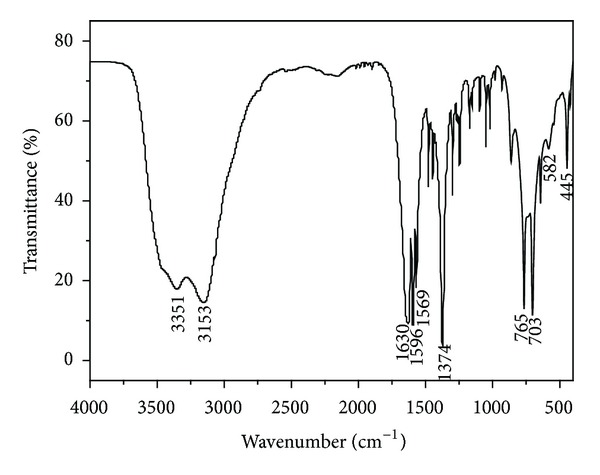
FT-IR spectra of the title complex.

The FT-IR spectra of 2-picolinic acid and the title complex are given in Figures 6 and 7. Comparison of IR spectra of the free ligand reveals that considerable changes in frequencies have occurred which can determine the coordination sites in chelation. A wide intense absorption band around 3351 cm^−1^ can be assigned to stretching vibration of hydroxyl from the water molecules. The bands corresponding to the stretching vibration of the C–H and C=N are situated at 3153 cm^−1^ and 1630 cm^−1^, respectively. The vibration peak found in the 1569 cm^−1^ region is assigned to the stretching vibration of the C=C–C=C bond. The difference value of 222 cm^−1^ between the asymmetric (1596 cm^−1^) and symmetric (1374 cm^−1^) stretching vibration of the carboxylate group is in line with a monodentate type of coordination [29–31]. The band corresponding to the stretching vibration of the C=O group of the Hpic monomer is situated at 1700–1769 cm^−1^ and disappears in the complex. The IR spectra of 2-picolinic acid contain broad absorption bands at 2607 cm^−1^ and 2152 cm^−1^ and indicate the existence of O–H*⋯*N type of intermolecular hydrogen bonding, but it disappears in the complex whose phenomenon confirms that the nitrogen atom is coordinated to the cobalt ion. The absorption peaks at 765 cm^−1^ and 703 cm^−1^ for the complex are assigned to deformation vibration of the pyridine ring and compared with the absorption peaks at 752 cm^−1^ and 684 cm^−1^ from the 2-picolinic acid ligand, which confirms that the pyridyl N atom and carboxyl O atom are coordinated with the center cobalt ion. The absorption peak found in the 445 cm^−1^ region is assigned to the Co–N bond and in the 422 cm^−1^ region is assigned to the Co–O bond [32].

### 3.3. Thermal Analysis

**Figure 8:**
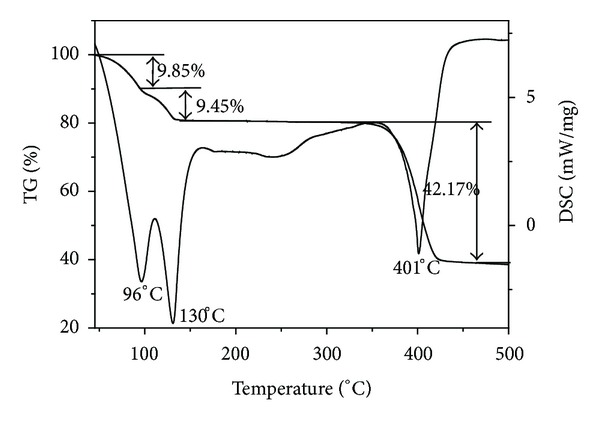
TG-DSC curves of the title complex.

**Figure 9:**
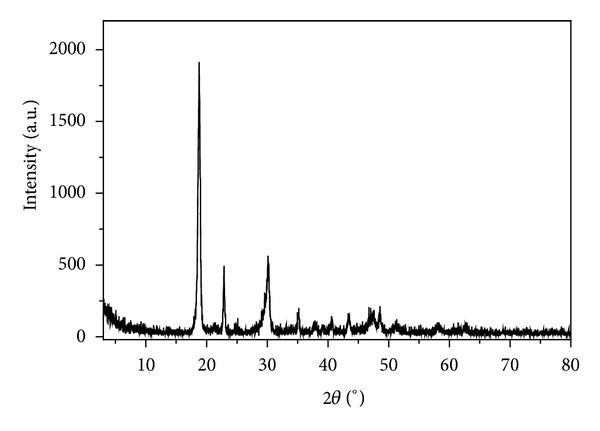
XRD pattern of the residue.

To study the thermal decomposition process of complexes is helpful to the understanding of the coordination structure of the complexes [33, 34]. The thermal stability of the title complex in nitrogen was investigated by TG-DSC analysis. The TG-DSC curves of the complex are shown in Figure 8, and the possible pyrolysis reaction and the experimental and calculated percentage mass losses in the thermal decomposition process of the complex are summarized in Table 4. As Figure 8 shows, there are three endothermic peaks at 96°C, 130°C, and 401°C in the DSC curve. The first mass loss of [Co(pic)_2_(H_2_O)_2_] · 2H_2_O ·2H_2_O occurs at about 96°C, corresponding to the release of two molecules of crystalline water. This is consistent with the single crystal structure. The experimental percentage mass loss (9.85%) is close to the calculated one (9.60%). The second mass loss of 9.45% (calcd. 9.60%) occurs between 100 and 140°C, which show loss of two water molecules from the complex. Because of the high dehydration temperature, this loss of the two water molecules should be coordinated water. After the four water molecules are lost, the complex become to be [Co(pic)_2_]. The residue complex of [Co(pic)_2_] is relatively stable in nitrogen between 140 and 350°C. The next step mass loss in the temperature ranges of 350–450°C corresponds to the loss of two pyridine free radicals (·C_5_H_4_N), and the two free radicals may eventually form 2,2′-bipyridine and then escape to volatile. This is just why there is an appreciable endothermic peak at 401°C in the DSC curve. The mass loss of 42.17% in the TG curve is in good agreement with the calculated result of 41.63%. The residual mass of the complex remains almost constant in nitrogen until 500°C. The composition of the final residue is cobalt oxalate, and the experimental result (39.17%) is in agreement with the result of theoretical calculation (38.53%). The XRD pattern of the residue is shown in Figure 9, and the characteristic peaks of the residue are mostly consistent with the normative peaks according to the JCPDS cards number 25-0250 (CoC_2_O_4_). The result of thermal analysis further ascertains that the molecule composition of the complex is [Co(pic)_2_(H_2_O)_2_] · 2H_2_O.

## 4. Conclusion

The complex [Co(pic)_2_(H_2_O)_2_] · 2H_2_O was synthesized with the reaction of cobalt acetate and 2-picolinic acid as the reactants. The composition and structure of the complex were characterized by EA, FTIR, single crystal X-ray diffraction, and TG-DSC. The crystal structure of the complex belongs to monoclinic system and space group *P*2(1)/*n*, with cell parameters of *a* = 9.8468(7) Å, *b* = 5.2013(4) Å, *c* = 14.6041(15) Å, *β* = 111.745(6)°, *Z* = 2, and *D*_*c*_ = 1.666 g  cm^−3^. The cobalt ion is six-coordinated by two pyridyl N atoms and two carboxyl O atoms from two 2-picolinic anions and two O atoms from two H_2_O molecules, forming a distorted octahedral geometry. The thermal decomposition processes of the complex under nitrogen include dehydration and pyrolysis of the ligand, and the final residue is cobalt oxalate at about 450°C.

## Figures and Tables

**Table 1:** Crystal data and structure refinement parameters for the title complex.

Empirical formula	CoC_12_H_16_O_8_N_2_	*F*(000)	386
Formula weight	375.20 g mol^−1^	Crystal size	0.42 mm × 0.35 mm × 0.25 mm
Temperature	298(2) K	Theta range for data collection	2.50–25.02°
Wavelength	0.71073 Å	Limiting indices	−11 ≤ *h* ≤ 8, −6 ≤ *k* ≤ 6, −15 ≤ *l* ≤ 17
Crystal system	Monoclinic	Reflections collected/unique	3424/1320 [*R*(int) = 0.0438]
Space group	*P*2(1)/*n *	Completeness to theta = 25.02	99.6%
Unit cell dimensions		Absorption correction	Semiempirical from equivalents
*a *	9.8468(7) Å	Max. and min. transmission	0.7549 and 0.6344
*b *	5.2013(4) Å	Refinement method	Full-matrix least squares on *F* ^2^
*c *	14.6041(15) Å	Data/restraints/parameters	1320/0/107
**β**	90.2310(10)°	Goodness of fit on *F* ^2^	1.077
Volume	747.96(11) Å^3^	Final *R* indices [*I* > 2*σ*(*I*)]	*R*_ 1_ = 0.0297, *wR*_2_ = 0.0831
*Z *	2	*R* indices (all data)	*R*_ 1_ = 0.0338, *wR*_2_ = 0.0877
Calculated density	1.666 g cm^−3^	Extinction coefficient	0.222(10)
Absorption coefficient	1.192 mm^−1^	Largest diff. peak and hole	0.333 and −0.358 e Å^−3^

**Table 2:** Selected bond lengths (Å) and angles (°) for the title complex.

Co(1)–O(1)#1	2.0765(15)	Co(1)–O(1)	2.0765(15)
Co(1)–N(1)#1	2.1196(18)	Co(1)–N(1)	2.1196(18)
Co(1)–O(3)#1	2.1477(16)	Co(1)–O(3)	2.1477(16)
N(1)–C(6)	1.339(3)	N(1)–C(2)	1.344(3)
O(1)–C(1)	1.277(3)	O(2)–C(1)	1.242(3)
O(1)#1–Co(1)–O(1)	180.00(8)	O(1)#1–Co(1)–N(1)#1	78.92(7)
O(1)–Co(1)–N(1)#1	101.08(7)	O(1)#1–Co(1)–N(1)	101.08(7)
O(1)–Co(1)–N(1)	78.92(7)	N(1)#1–Co(1)–N(1)	180.0
O(1)#1–Co(1)–O(3)#1	90.89(6)	O(1)–Co(1)–O(3)#1	89.11(6)
N(1)#1–Co(1)–O(3)#1	84.49(7)	N(1)–Co(1)–O(3)#1	95.51(7)
O(1)#1–Co(1)–O(3)	89.11(6)	O(1)–Co(1)–O(3)	90.89(6)
N(1)#1–Co(1)–O(3)	95.51(7)	N(1)–Co(1)–O(3)	84.49(7)
O(3)#1–Co(1)–O(3)	180.0	C(2)–N(1)–Co(1)	112.10(15)
C(1)–O(1)–Co(1)	116.23(14)	O(1)–C(1)–C(2)	116.10(19)
N(1)–C(2)–C(1)	116.00(18)	O(2)–C(1)–O(1)	124.8(2)
O(2)–C(1)–C(2)	119.1(2)		

Symmetry transformations used to generate equivalent atoms: #1 – *x* + 1, –*y* + 1, −*z* + 1.

**Table 3:** Hydrogen bond lengths (Å) and bond angles (°) for the title complex.

D–H	*d* (D–H)	*d* (H*⋯*A)	∠DHA	*d* (D*⋯*A)	A
O3–H3C	0.850	1.896	171.34	2.739	O1 [–*x* + 1, −*y*, −*z* + 1]
O3–H3D	0.851	1.963	171.40	2.808	O4 [*x* − 1/2, −*y* + 3/2, *z* + 1/2]
O4–H4C	0.850	1.852	175.51	2.700	O2 [*x*, *y* + 1, *z *]
O4–H4D_a	0.850	2.095	175.29	2.943	O4 [−*x* + 3/2, *y* – 1/2, −*z* + 1/2]
O4–H4B_b	0.849	2.203	145.57	2.943	O4 [−*x* + 3/2, *y* + 1/2, −*z* + 1/2]

**Table 4:** Thermal decomposition data of the title complex.

Reaction	DSC/°C	Mass loss %	
		*W*_exp_	*W*_cal_
[Co(C_5_H_4_NCOO)_2_(H_2_O)_2_]·2H_2_O			
↓−2H_2_O	96 (endo.)	9.85	9.60
[Co(C_5_H_4_NCOO)_2_(H_2_O)_2_]			
↓−2H_2_O	130 (endo.)	9.45	9.60
[Co(C_5_H_4_NCOO)_2_]			
↓−C_5_H_4_N–C_5_H_4_N (2,2′-bipy)	401 (endo.)	42.17	41.63
CoC_2_O_4_		38.53*	39.17**

*The experimental percentage mass of the residue in the sample.

**The calculated percentage mass of the residue in the sample.

## Additional Material

Crystallographic data for the title complex [Co(pic)_2_(H_2_O)_2_] · 2H_2_O has been deposited with the Cambridge Crystallographic Data Centre. The deposition number is CCDC-913361. The data can be obtained free of charge on application to the Director, CCDC, 12 Union Road, Cambridge, UK; fax: +44 1223 366 033; e-mail: deposit@ccdc.ac.uk, or on the web http://www.ccdc.cam.ac.uk/pages/Home.aspx, or from the authors on request.

## References

[1] Li YW, Tao Y, Hu TL (2012). Synthesis, structure, and photoluminescence of Zn(II) and Cd(II) coordination complexes constructed by structurally related 5, 6-substituted pyrazine-2, 3-dicarboxylate ligands. *Solid State Sciences*.

[2] Colacio E, María A. Palacios MAP, Antonio Rodríguez-Díeguez AR (2010). 3d-3d-4f chain complexes constructed using the dinuclear metallacyclic complex [Ni_2_(mbpb)_3_]^2-^[H_2_mbpb = 1, 3-Bis(pyridine-2-carboxamide)benzene] as a ligand: synthesis, structures, and magnetic properties. *Inorganic Chemistry*.

[3] Günay G, Yeşilel OZ, Soylu MS, Keskin S, Dal H (2011). Two novel 2D and 3D coordination polymers constructed from pyrazine-2,3-dicarboxylic acid and chloride bridged secondary building units. *Synthetic Metals*.

[4] Cui J, Li Y, Guo Z, Zheng H (2013). A porous metal-organic framework based on Zn_6_O_2_ clusters: chemical stability, gas adsorption properties and solvatochromic behavior. *Chemical Communications*.

[5] Fang SM, Sañudo EC, Hu M (2011). Hydrothermal syntheses, crystal structures, and magnetic properties of a series of unique three-dimensional lanthanide-organic coordination frameworks with a *N*-protonated 2,6-dihydroxypyridine-4-carboxylate tecton. *Crystal Growth and Design*.

[6] Yang Q, Zhao J, Hu B, Zhang X, Bu X (2010). New manganese(II) azido coordination polymers with nicotinic/isonicotinic acids as coligands: synthesis, structure, and magnetic properties. *Inorganic Chemistry*.

[7] Hu X, Zeng Y, Chen Z (2009). 3d-4f coordination polymers containing alternating EE/EO azido chain synthesized by synergistic coordination of lanthanide and transition metal ions. *Crystal Growth and Design*.

[8] Tanner PA, Duan C (2010). Luminescent lanthanide complexes: selection rules and design. *Coordination Chemistry Reviews*.

[9] Huang YG, Jiang FL, Hong MC (2009). Magnetic lanthanide-transition-metal organic-inorganic hybrid materials: from discrete clusters to extended frameworks. *Coordination Chemistry Reviews*.

[10] Cui GH, He CH, Jiao CH, Geng JC, Blatov VA (2012). Two metal-organic frameworks with unique high-connected binodal network topologies: synthesis, structures, and catalytic properties. *CrystEngComm*.

[11] Novitski G, Borta A, Shova S, Kazheva ON, Gdaniec M, Simonov YA (2008). Synthesis and structure of Co(III) complexes with 2-pyridinecarboxylic acid. *Russian Journal of Inorganic Chemistry*.

[12] Kukovec BM, Popović Z, Pavlović G, Rajić Linarić M (2008). Synthesis and structure of cobalt(II) complexes with hydroxyl derivatives of pyridinecarboxylic acids: conformation analysis of ligands in the solid state. *Journal of Molecular Structure*.

[13] Luo JH, Hong MC, Shi Q (2002). Synthesis, structure and magnetic properties of a quasi-two-dimensional compound [Cu(C_5_H_4_NCOO)_2_]*·*2H_2_O. *Transition Metal Chemistry*.

[14] Vargová Z, Zeleòák V, Císaøová I, Györyová K (2004). Correlation of thermal and spectral properties of zinc(II) complexes of pyridinecarboxylic acids with their crystal structures. *Thermochimica Acta*.

[15] Zhong GQ, Zhong WW, Jia RR, Jia YQ (2013). Solid-state synthesis, characterization and biological activity of the bioinorganic complex of aspartic acid and arsenic triiodide. *Journal of Chemistry*.

[16] Zhong GQ, Jia RR, Jia YQ (2012). Solid-solid reaction preparation for nanoparticles of bioinorganic complex of bismuth and serine at room temperature. *Advanced Materials Research*.

[17] Min CY, Yang XF, Zhang RX, Yao F, Ouyang WM (2011). Greener solid state synthesis of a ternary lanthanum complex at room temperature. *Journal of Coordination Chemistry*.

[18] Méret M, Bienz S (2008). Efficient and flexible solid-phase synthesis of *N*-hydroxypolyamine derivatives. *European Journal of Organic Chemistry*.

[19] Kukovec BM, Kodrin I, Mihalić Z, Furić K, Popović Z (2010). Cis-trans isomerism in cobalt(II) complexes with 3-hydroxypicolinic acid. Structural, DFT and thermal studies. *Inorganica Chimica Acta*.

[20] Failes TW, Cullinane C, Diakos CI, Yamamoto N, Lyons JG, Hambley TW (2007). Studies of a cobalt(III) complex of the MMP inhibitor marimastat: a potential hypoxia-activated prodrug. *Chemistry*.

[21] López-Sandoval H, Londoño-Lemos ME, Garza-Velasco R (2008). Synthesis, structure and biological activities of cobalt(II) and zinc(II) coordination compounds with 2-benzimidazole derivatives. *Journal of Inorganic Biochemistry*.

[22] Dimiza F, Papadopoulos AN, Tangoulis V (2010). Biological evaluation of non-steroidal anti-inflammatory drugs-cobalt(ii) complexes. *Dalton Transactions*.

[23] Yeşilel OZ, Mutlu A, Darcan C, Büyükgüngör O (2010). Syntheses, structural characterization and antimicrobial activities of novel cobalt-pyrazine-2,3-dicarboxylate complexes with *N*-donor ligands. *Journal of Molecular Structure*.

[24] Segl’a P, Miklovič J, Mikloš D (2008). Crystal structure, spectroscopic and magnetic properties, and antimicrobial activities of cobalt(II) 2-methylthionicotinate complexes with *N*-heterocyclic ligands. *Transition Metal Chemistry*.

[25] Georgiou D, Papangelakis VG (2009). Behaviour of cobalt during sulphuric acid pressure leaching of a limonitic laterite. *Hydrometallurgy*.

[26] Sheldrick GM (1997). *SHELXS 97, Program for the Solution of Crystal Structure*.

[27] Sheldrick GM (1997). *SHELXS 97, Program for the Refinement of Crystal Structure*.

[28] Janiak C (2000). A critical account on *π* − *π* stacking in metal complexes with aromatic nitrogen-containing ligands. *Journal of the Chemical Society, Dalton Transactions*.

[29] Neelakantan MA, Rusalraj F, Dharmaraja J, Johnsonraja S, Jeyakumar T, Sankaranarayana Pillai M (2008). Spectral characterization, cyclic voltammetry, morphology, biological activities and DNA cleaving studies of amino acid Schiff base metal(II) complexes. *Spectrochimica Acta A*.

[30] Zhong G, Shen J, Jiang Q, Yu K (2011). Synthesis and structural determination of a novel heterometallic complex [Sb_2_(edta)_2_-*μ*
_4_-Co(H_2_O)_2_]*·*5.15H_2_O. *Chinese Journal of Chemistry*.

[31] Wang J, Zhang X, Liu Z, Jia W (2002). Synthesis and structural determination of binuclear nine-coordinate (NH_4_)_4_[Yb_2_(dtpa)_2_] *·*9H_2_O. *Journal of Molecular Structure*.

[32] Nakamoto K (1986). *Infrared and Raman Spectra of Inorganic and Coordination Compounds*.

[33] Zhong GQ, Shen J, Jiang QY, Jia YQ, Chen MJ, Zhang ZP (2008). Synthesis, characterization and thermal decomposition of Sb^III^-M-Sb^III^ type trinuclear complexes of ethylenediamine-*N, N, N’, N’*-tetraacetate (M:Co(^II^), La(^III^), Nd(^III^), Dy(^III^)). *Journal of Thermal Analysis and Calorimetry*.

[34] Allan JR, Geddes WC, Hindle CS, Orr AE (1989). Thermal analysis studies on pyridine carboxylic acid complexes of zinc(II). *Thermochimica Acta C*.

